# Redundant Mechanisms Prevent Mitotic Entry Following Replication Arrest in the Absence of Cdc25 Hyper-Phosphorylation in Fission Yeast

**DOI:** 10.1371/journal.pone.0021348

**Published:** 2011-06-23

**Authors:** Corey Frazer, Paul G. Young

**Affiliations:** Department of Biology, Queen's University, Kingston, Ontario, Canada; University of Calgary, Canada

## Abstract

Following replication arrest the Cdc25 phosphatase is phosphorylated and inhibited by Cds1. It has previously been reported that expressing Cdc25 where 9 putative amino-terminal Cds1 phosphorylation sites have been substituted to alanine results in bypass of the DNA replication checkpoint. However, these results were acquired by expression of the phosphorylation mutant using a multicopy expression vector in a genetic background where the DNA replication checkpoint is intact. In order to clarify these results we constructed a Cdc25(9A)-GFP native promoter integrant and examined its effect on the replication checkpoint at endogenous expression levels. In this strain the replication checkpoint operates normally, conditional on the presence of the Mik1 kinase. In response to replication arrest the Cdc25(9A)-GFP protein is degraded, suggesting the presence of a backup mechanism to eliminate the phosphatase when it cannot be inhibited through phosphorylation.

## Introduction

Faithful DNA replication and chromosome segregation is critical for cell viability. A universally conserved checkpoint exists in eukaryotes which prevents mitotic initiation while DNA is being replicated. Failure of this checkpoint has catastrophic consequences for the cell including chromosome loss and ultimately cell death [1], [2].

In *Schizosaccharomyces pombe*, progression though the G2/M transition is dependent on the phosphorylation state of tyrosine 15 (Y15) of the Cdc2 cyclin dependent kinase [3], [4]. Wee1 and Mik1 kinases are responsible for the inhibitory Cdc2-Y15 phosphorylation [5], [6]–[8]. Cdc2 is dephosphorylated by the Cdc25 phosphatase, causing Cdc2 activation and mitotic entry [9]–[11]. A second phosphatase, Pyp3, is able to dephosphorylate Cdc2 in vitro and rescue loss of *cdc25* when overexpressed. Pyp3 is essential in cells lacking both Cdc25 and Wee1 [12].

Cdc25 expression is cell cycle regulated, accumulating through G2 and reaching its peak as the cell enters mitosis and then returning to basal levels in G1 and S-phase [13], [14]. This is accomplished through a combination of oscillating mRNA levels and proteolysis [14], [15]. Cdc25 is imported into the nucleus via the importin-β Sal3 [16].

Following DNA damage and replication arrest the Chk1 and Cds1 kinases negatively regulate mitotic entry by phosphorylating Cdc25 [17]–[19]. These phosphorylations create binding sites for the 14-3-3 protein, Rad24, resulting in export from the nucleus to the cytoplasm. In fission yeast, Wee1 is phosphorylated by both Cds1 in response to replication blocks [17] and Chk1 in response to DNA damage [20]. However, the phosphorylation of Wee1 does not affect its Cdc2-Y15 phosphorylation activity in vitro [21]. Mik1 tyrosine kinase plays only a minor role in the regulation of Cdc2 activity during G2 [6] but is involved in preventing mitotic entry following replication arrest [22].

The DNA damage and DNA replication checkpoints have several proteins in common that signal to the effector kinases Cds1 and Chk1. Rad1, Hus1 and Rad9 form a heterotrimer (9-1-1 complex) which forms a ring structure around the double helix similar to that of the proliferating cell nuclear antigen (PCNA). The ATM (Ataxia-Telangiectasia Mutated) homologue Rad3 phosphorylates and activates Cds1 or Chk1 depending on the cell cycle stage and nature of the upstream signal [23], [24]. Cds1 and Chk1 require adapter proteins Mrc1 and Crb1, respectively, for Rad3 interaction [25]–[28]. Since the DNA damage and DNA replication checkpoints utilize a number of the same upstream components; bifurcation of the pathway in response to different stimuli is required. This is primarily accomplished by restriction of Cds1 and Mrc1 expression to S-phase [28], [29].

In addition to inhibiting the G2/M transition Cds1 functions to prevent DNA recombination at stalled replication forks by phosphorylating Holiday Junction resolvase subunit Mus81 [30]–[32], double strand break repair protein Rad60 [33], and the RecQ-family helicase Rqh1 [34], [35]. Cds1 activation results in the phosphorylation and inhibition of Nrm1, a transcriptional repressor of the Cdc10-Res2 complex which regulates the G1 transcription of genes containing *MluI*-box elements in their promoters [36]–[38]. Nrm1 targets include ribonucleotide reductase subunit Cdc22 [39], [40] and the Cdc2 kinase Mik1 [41]. Cds1 also phosphorylates Clp1/Flp1 phosphatase [42] the *S. pombe* CDC14 homologue involved in actomyosin ring stability, cytokinesis and mitotic exit [43]–[47]. In addition, Clp1/Flp1 has been shown to dephosphorylate the Cdc2 targeted S/TP sites on Cdc25, although the precise identity of these sites has yet to be determined [15].

Although Cdc25 is phosphorylated, interacts with Rad24, and is exported from the nucleus following DNA damage or replication blocks [48] it is not certain which of these steps are essential for checkpoint function. Cytoplasmic Cdc25 localization appears to be dispensable since forcing Cdc25 into the nucleus with addition of an SV-40 NLS sequence does not override the checkpoint [49]. The question of whether Cdc25 phosphorylation and Rad24 binding are required for the DNA replication checkpoint was addressed by Zeng and Piwnica-Worms [50], who mutated nine in vitro Cds1 serine/threonine phosphorylation sites to alanine, creating Cdc25(9A). When introduced into the cell on a multicopy plasmid under the control of an attenuated *nmt1* promoter this construct caused bypass of the DNA replication checkpoint. They concluded that Cdc25 phosphorylation on at least some of those sites was required for proper DNA replication checkpoint function.

We have re-examined these findings and show that the results of the previous work with Cdc25(9A) were influenced by overexpression of the phosphorylation site mutant protein. When expressed under the control of its native promoter as a single-copy chromosomal integrant the DNA replication checkpoint is functional. Under these conditions the replication checkpoint is maintained through the action of Mik1 and is not dependent on these Chk1 phosphorylation sites on Cdc25. In addition, the Cdc25(9A)-GFP protein is degraded following checkpoint activation, suggesting that inhibition of Cdc25 by the replication checkpoint is reinforced by degrading any Cdc25 which is not phosphrorylated and/or 14-3-3 bound.

## Results

### Creation of *cdc25-GFPint* and *cdc25(9A)-GFPint* native promoter integrants

**Figure 1:**
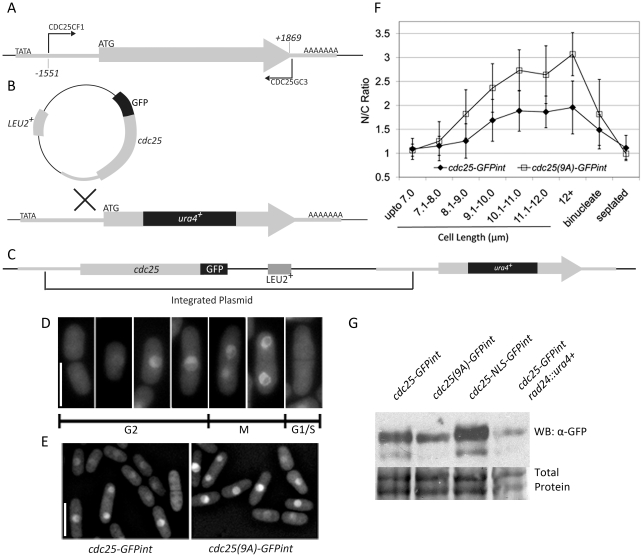
Cell cycle dependent localization of Cdc25. **A.** PCR amplification of Cdc25 and upstream sequence. **B.** Integration of *pcdc25-GFP* plasmid into the native cdc25 promoter by homologous recombination. **C.** Post-integration structure of *cdc25-GFPint* strain. **D.** Logarithmically growing cells expressing Cdc25-GFP from the chromosomal *cdc25* promoter in YEA imaged by fluorescence microscopy. Bar represents 10 µm. **E.** Fluorescence images of Cdc25-GFP and Cdc25(9A)-GFP at mid-logarithmic phase in YEA at 30°C. Logarithmic phase 30°C cultures imaged using fluorescence microscopy. Bar represents 10 µm. **F.** Nuclear∶Cytoplasmic ratio of Cdc25-GFP and Cdc25(9A)-GFP fluorescence in logarithmically growing populations of cells. (n = 200. Each size category contains 20 to 40 cells. Error bars represent ±1 s.d. from the mean). **G.** Western blot comparing the relative Cdc25-GFP levels in the strains indicated. Cultures were grown to mid-log phase, harvested, lysed and analysed by SDS-PAGE and western blotting using mouse anti-GFP primary and anti-mouse HRP secondary antibodies.

In order to examine the localization and regulation of Cdc25 under native expression levels the *cdc25^+^* and *cdc25(9A)* open reading frames were fused to GFP and integrated at the endogenous *cdc25^+^* locus in a strain bearing the disrupted *cdc25::ura4^+^* allele. The *cdc25^+^* ORF and 1551 base pairs of upstream sequence were amplified by PCR (Figure 1A). This fragment was ligated into the *pREP1-GFP* plasmid from which the 1200 bp *nmt1* promoter had been removed by digestion with *PstI* and *SalI* (Figure 1B). This plasmid was integrated into the *S. pombe* genome by a single crossover at the *cdc25^+^* locus in a *cdc25::ura4^+^ cdc2-3w ura4-D18 leu1-32* background (Q1975, Figure 1C). The strain was then out-crossed to remove the *cdc2-3w* mutation. To create a *cdc25(9A)-GFP* integration plasmid, *pREP81-cdc25(9A)* [50] was digested with *SplI* and *BglI* to liberate a fragment containing all 9 of the S/T to A substitutions. This fragment was then ligated into *SplI/BglI* digested *cdc25^+^* integration plasmid and the resulting construct integrated at the native *cdc25^+^* locus as described above. The structure of these integrations was confirmed by Southern hybridization using ^32^P-labeled *cdc25^+^* as a probe (data not shown). The *cdc25::ura4^+^* knockout, although clearly non-functional, left a portion of the COOH-terminus of Cdc25 intact, including the entire catalytic domain [9]. To ensure that this domain does not alter the phenotype of our native promoter integrant constructs, the entire *ura4^+^* disrupted *cdc25^+^* ORF was replaced with a *kan-MX6* cassette in *cdc25-GFPint* and *cdc25(9A)-GFPint*. *cdc25-GFPint* and *cdc25(9A)-GFP* strains in the *cdc25::kanMX6* background were tested in several experiments including the functioning of the checkpoints in a *mik1::ura4^+^* background (see below) they were indistinguishable from strains where endogenous *cdc25* was disrupted with a *ura4^+^* cassette (data not shown).

The native promoter driven *cdc25-GFP* fusion integrant (Q2016 *cdc25-GFPint*) divides at a wildtype length without any apparent effect on cell cycle timing. In rich media, *cdc25-GFPint* and wildtype divide at 13.08 µm±1.04 and 13.25 µm±0.99 respectively (n = 100, Student's t-test; *p* = 0.253). In minimal media (EMM) *cdc25-GFPint* and wildtype divide at 13.02±0.89 and 13.20±0.97 µm, respectively (n = 100, Student's t-test; *p* = 0.164). Cdc25-GFP accumulates in the nucleus as G2 cells progress towards the G2/M transition, and disappears as cells complete mitosis and undergo septation (Figure 1D).

Cells expressing *cdc25(9A)-GFPint* (Q3792), lacking 9 in vitro Cds1 phosphorylation sites, are not significantly different in length from *cdc25-GFPint* (12.72±0.80 µm and 12.87±1.0 µm respectively, n = 30, Student's t-test; *p* = 0.498). The localization pattern of Cdc25(9A)-GFP resembles that of Cdc25-GFP but Cdc25(9A)-GFP accumulates to significantly higher levels in the cell nuclei than Cdc25-GFP in all G2 size classes (Student's t-test; *p*<0.05) (Figure 1E and F). This result is in contrast to the clear mitotic advancement that occurs when Cdc25 is made constitutively nuclear by addition of the SV-40 NLS [16], [49], or deletion of *rad24* [48], [51], both of which also result in enhanced nuclear localization of Cdc25. Although *cdc25-NLS-GFPint* and *cdc25-GFPint rad24::ura4^+^* divide at a similar size, the level of Cdc25 protein differs greatly (Figure 1G). Cdc25-NLS-GFP is present at significantly increased levels relative to Cdc25-GFP, which could perhaps account for a smaller size at mitotic entry. However while loss of *rad24* results in enhanced nuclear localization of Cdc25 it does not affect its overall protein concentration.

### *cdc25(9A)-GFPint* has increased sensitivity to replication blocks and DNA damage

**Figure 2:**
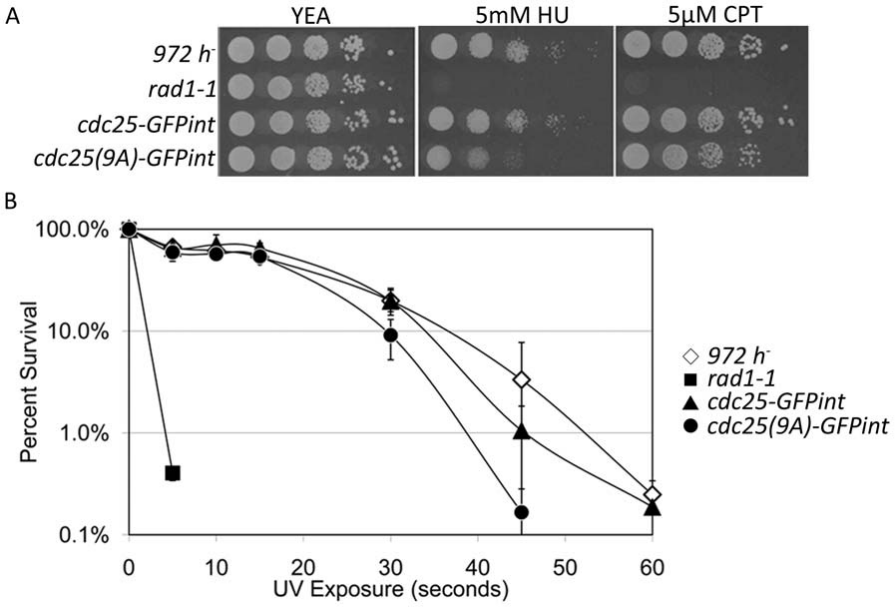
Sensitivity of *cdc25(9A)-GFPint* to replication blocks and DNA damage. **A.** Logarithmically growing cultures were diluted to 1×10^6^ then serially diluted 1∶10 onto YEA, YEA containing 5 mM hydroxyurea or YEA containing 5 µM camptothecin and incubated for 3 days at 30°C. **B.** Percent survival following UV exposure. Log cultures serially diluted, plated on yeast extract media and exposed to UV light (560 µW/cm^2^). Error bars represent ± s.d. of the results of 3 independent experiments.

To examine the effect of loss of Cds1 phosphorylation sites on Cdc25 function the sensitivity of *cdc25(9A)-GFPint* (Q3792) to DNA damage and replication arrest was examined by exposing cells to hydroxyurea, camptothecin and UV irradiation. Hydroxyurea (HU) is a ribonucleotide reductase inhibitor which causes a DNA replication checkpoint arrest due to depletion of intracellular dNTP pools [52]. Camptothecin (CPT) is a topoisomerase (Top1) inhibitor which causes covalent Top1-DNA linkages and double strand breaks [53]. This results in DNA damage in late S-phase, Chk1 activation and a DNA damage checkpoint arrest [54], [55]. When cultured for several days on plates containing HU, growth of *cdc25(9A)-GFPint* is reduced relative to that of *cdc25-GFPint*, although it is not nearly as severely affected as the checkpoint deficient mutant *rad1-1* (Figure 2A). Following release from arrest in liquid culture containing 15 mM HU, *cdc25(9a)-GFPint* looses approximately 30 percent of its viability over the course of 8 hours (data not shown). In a similar experiment, CPT was shown to have a marginal effect on the growth of *cdc25(9A)-GFPint*. *cdc25-GFPint* behaves identically to wildtype in each of these experiments indicating that addition of the GFP tag to Cdc25 does not affect checkpoint function. *cdc25(9A)-GFPint* is modestly more sensitive to UV than *cdc25-GFPint* or wildtype (Figure 2B).

### *cdc25(9A)-GFPint* has an intact DNA replication checkpoint

To compare the HU sensitivity of cells expressing *cdc25(9A)-GFP* from the native promoter to those expressing it from the *pREP81 plasmid* expressed in a *cdc25-22^ts^* background, the “cut” accumulation experiment of Zeng and Piwnica-Worms [50] was replicated. To allow western blot analysis of protein levels using commercial anti-GFP antibodies the *cdc25(9A)* ORF was cloned into *pREP81-GFP*.

**Figure 3:**
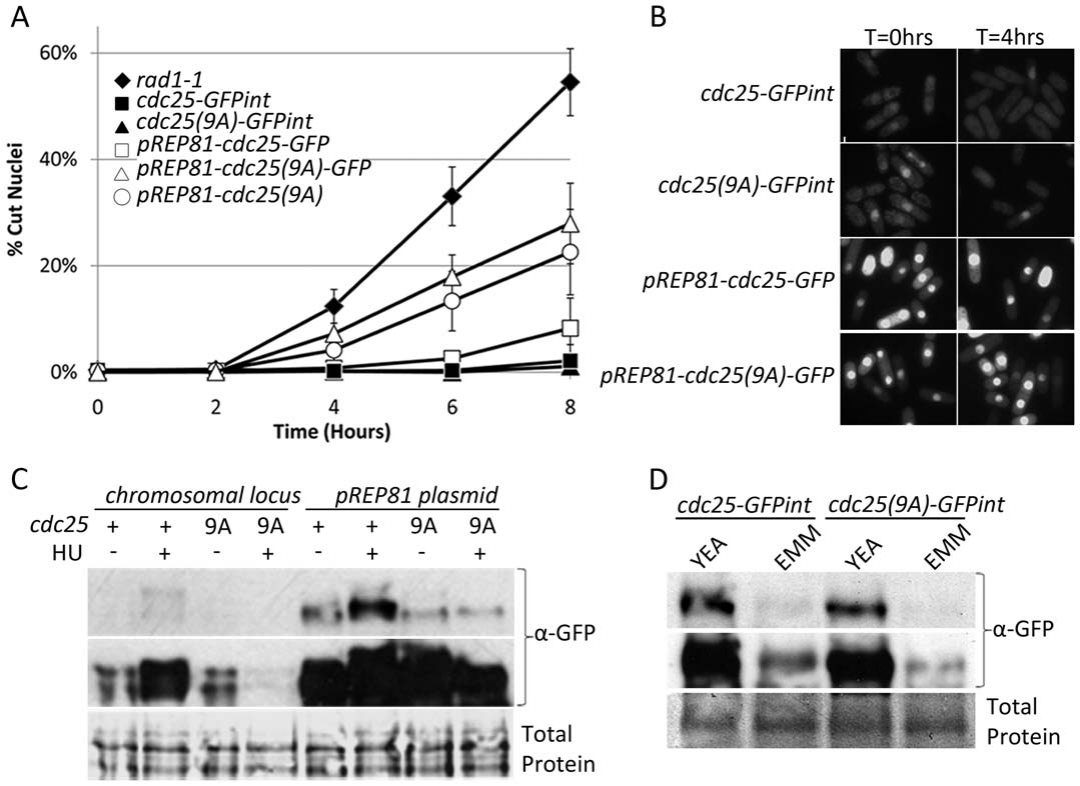
Replication checkpoint proficiency of Cdc25(9A)-GFP native promoter integrants. **A.** Checkpoint sensitivity to HU treatment. Logarithmically growing cultures were exposed to 15 mM hydroxyurea. Samples were methanol fixed at two hour intervals, DAPI stained and examined for cut phenotypes. Error bars represent ±1 s.d. of the mean percent cut phenotype from three independent experiments. In each experiment at least four fields of 50–200 cells for each time point. **B, C.** GFP fluorescence of strains treated with HU in EMM for four hours. Logarithmically growing cultures were harvested by centrifugation prior to, and four hours after, exposure to 15 mM HU and photographed. Bar represents 10 µm **B** or processed for SDS-PAGE electrophoresis and western blotting **C** using mouse anti-GFP primary and anti-mouse HRP secondary antibodies. A longer exposure of the same membrane is represented in the center panel. The membrane was subsequently stained with Coomassie Brilliant Blue to show total protein (bottom panel). **D.** Cdc25-GFP and Cdc25(9A)-GFP protein levels in logarithmically growing cultures in either rich (YEA) or minimal (EMM) media. Top Panel: anti-GFP western blot. Middle Panel: Longer exposure. Bottom Panel: Membrane stained to show total protein.

Cells transformed with *pREP81-cdc25(9A)-GFP* showed a significantly greater accumulation cut phenotypes than those containing *pREP81-cdc25-GFP*. Addition of the GFP tag does not affect the accumulation of cut phenotypes in strains expressing *cdc25(9A)* from the *pREP81* plasmid (Student's t-test *p* = 0.35, 0.41 and, 0.52 at T = 4, 6, 8 hours respectively). Native promoter expressed *cdc25-GFPint* and *cdc25(9A)-GFPint* strains show an extremely low frequency of cut phenotypes even after 8 hours of hydroxyurea exposure (Figure 3A). Examination of GFP fluorescence (Figure 3B) and anti-GFP western blots (Figure 3C) show that *pREP81* expression of Cdc25 exceeds that of the native promoter construct by approximately 10 fold.

Western blot analysis of lysates from cells expressing Cdc25-GFP and Cdc25(9A)-GFP from the native promoter shows that these two proteins are present in the cells at roughly equivalent concentrations (Figure 3C). Cdc25-GFP on either the native promoter or the attenuated *nmt1* promoter in the *pREP81* plasmid shows a decrease in electrophoretic mobility and accumulation of Cdc25 following HU arrest (Figure 3C). This is consistent with the stockpiling effect which has been described previously [56]. Cdc25(9A)-GFP expressed from the native promoter, rather than accumulating following HU treatment, is degraded. Thus, Cdc25 does not accumulate when the protein cannot be inhibited by Cds1 phosphorylation and/or by 14-3-3 binding. When expressed from the *pREP81* plasmid Cdc25(9A)-GFP is detectable at approximately the same level as before HU treatment and does not undergo a mobility shift. Therefore, the checkpoint defect of *pREP81-cdc25(9A)* may be due to the expression level of this protein exceeding the ability of the cell to degrade it. Thus, when replication is arrested in these cells there is still sufficient Cdc25 phosphatase activity present to cause bypass of the replication checkpoint and mitotic entry.

Use of *pREP81* requires minimal media for plasmid selection and for induction of the *nmt* promoter. Cdc25 translation is very sensitive to nutrient availability due to features in the 5′ un-translated region of the mRNA [57]. Western blot analysis of protein lysates shows that Cdc25 accumulates to a level approximately 10 fold lower in EMM than in YEA (Figure 3D). Interestingly, cells divide at the same length in minimal and rich media (data not shown). For ease of Cdc25-GFP detection, subsequent experiments were conducted using YEA media unless otherwise stated.

### Cdc25(9A)-GFP is unstable following activation of DNA replication checkpoint

**Figure 4:**
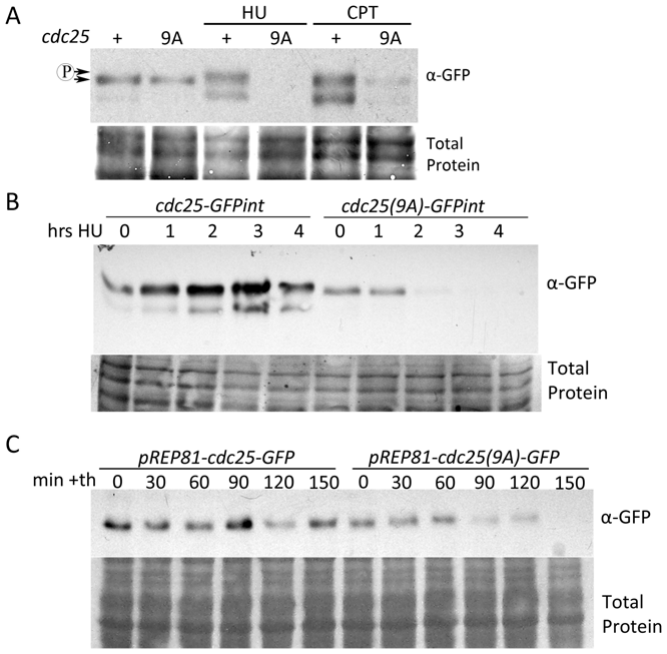
Cdc25(9A)-GFP is unstable following HU treatment and during logarithmic growth. **A.** Cdc25(9A)-GFP fails to be stockpiled following exposure to HU or CPT. Logarithmically growing cultures were exposed to 15 mM HU or 5 µM CPT for 4 hours prior to electrophoresis and western blot analysis using mouse anti-GFP primary antibody, anti-mouse HRP secondary. **B.** Cdc25(9A)-GFP is rapidly degraded following HU exposure. **C.** Cdc25(9A)-GFP is less stable than Cdc25-GFP during unperturbed growth. Cultures containing either *pREP81-cdc25-GFP* or *pREP81-cdc25(9A)-GFP* in *cdc25-22^ts^* were grown to mid logarithmic phase in EMM and their *nmt* promoters repressed with 20 µM thiamine. Samples were taken at 30 minute intervals and subjected to SDS-PAGE, and western blot analysis using mouse anti-GFP and anti-mouse-HRP antibody.

Using cdc^−^ mutants with various restriction points Kovelman and Russell [56] showed that Cdc25 continues to accumulate following cell cycle arrest; this effect can be seen in cells arrested in G1, S, G2 or during M-phase. Cdc25 also accumulates following HU exposure, but is maintained in an inactive form. This stockpiling may be adaptive in that it would allow rapid re-entry into the cell cycle once the checkpoint arrest is lifted. Western blot analysis of *cdc25-GFPint* and *cdc25(9A)-GFPint* following four hours of HU treatment shows that Cdc25(9A)-GFP is not stockpiled following a replication block (Figure 4A). Exposure of the same strains to 5 µM CPT in liquid culture demonstrates that Cdc25(9A)-GFP fails to be stockpiled following DNA damage checkpoint activation, but is not destabilized as seen following HU arrest.

Monitoring Cdc25 and Cdc25(9A) levels following HU exposure shows that Cdc25(9A)-GFP degradation is complete between one and two hours after addition of the drug (Figure 4B). In asynchronous culture only about ten percent of the population is undergoing S-phase at any particular time [58]. Those cells undergoing mitosis would come upon the replication block prior to cytokinesis as S-phase overlaps septum formation. By two hours the majority of cells which were in G2 at T = 0 would be through mitosis and arrested in S-phase with low Cdc25 levels. As this arrest proceeds Cdc25 normally accumulates [56]. The Cdc2/Cdc13 complex is localized to the nucleus for the duration of HU arrest [59]. Therefore, it is crucial that Cdc25 be negatively regulated in order to prevent mitotic entry. Cdc25 is degraded late in mitosis, via ubiquitination by the Anaphase Promoting Complex (APC) [60]. Thus, it is possible that Cdc25(9A)-GFP is prevented from accumulating in the S-phase following HU exposure because of sustained APC signaling. The mitotic exit phosphatase Clp1/Flp1 has recently been identified as a Cds1 target [42] and as a regulator of Cdc25 stability [15].

To determine the relative stability of Cdc25-GFP and Cdc25(9A)-GFP in logarithmically growing cultures, *pREP81-cdc25-GFP* and *pREP81-cdc25(9A)-GFP* cells were induced by culturing in minimal media lacking thiamine for 36 hours, followed by transcriptional repression by addition of 20 µM thiamine. Samples were taken every 30 minutes for 2.5 hours and analyzed by western blot (Figure 4C). Cdc25(9A)-GFP is clearly less stable than Cdc25-GFP under these conditions as Cdc25(9A)-GFP decreases to nearly undetectable levels after 2.5 hours of *nmt* promoter repression, while Cdc25-GFP is still abundant.

### Replication checkpoint arrest in Cdc25 phosphorylation mutants is maintained by Mik1

**Figure 5:**
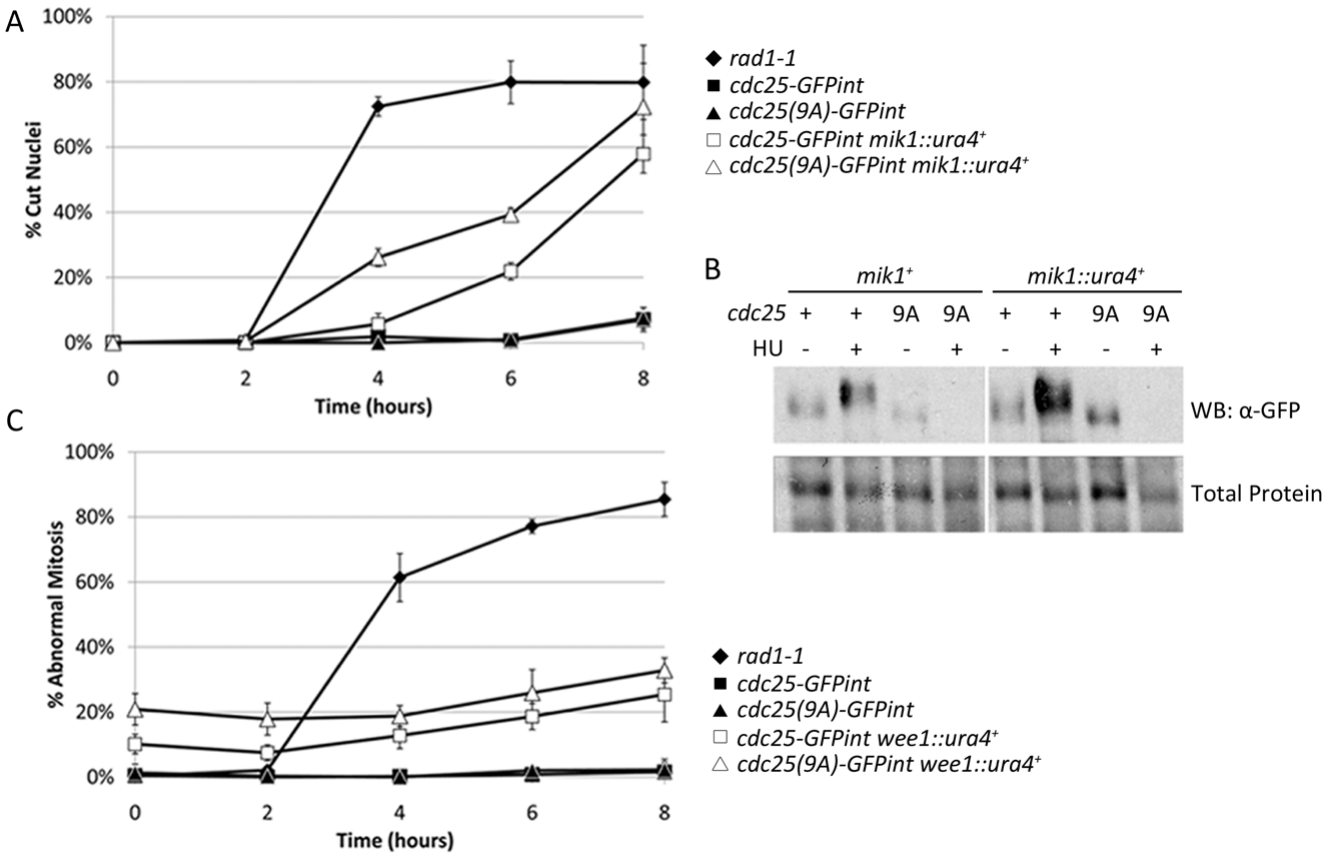
Mik1 and Wee1 are required for DNA replication checkpoint in cells expressing Cdc25(9A)-GFP or Cdc25(12A)-GFP. **A.** Mik1 is primarily responsible for enforcement of the replication checkpoint. Logarithmically growing cultures at 30°C were sampled prior to and at two hour intervals following addition of 15 mM HU. Samples were methanol fixed, DAPI stained and fields of cells photographed and examined for *cut* phenotypes. Error bars represent ±1 s.d. from the mean percent cut phenotype of at least four images containing 50–200 cells from one independent experiment. **B.** Cultures indicated were grown to mid-log phase and exposed to 15 mM HU for 4 hours before collection and processing. Samples were analyzed by SDS-PAGE and western blotting using mouse anti-GFP primary antibody, anti-mouse HRP secondary. **C.** Wee1 is a minor contributor to cell cycle arrest following HU exposure. Samples prepared as in “**A**”. Error bars represent ±1 s.d. from the mean percent cut phenotype of at least 4 images containing 50–200 cells from one independent experiment.

During a replication block, the Cdc2-Y15 kinase Mik1 participates in maintaining S-phase arrest [22]. Mik1 protein levels oscillate, peaking during S-phase. Mik1 nuclear accumulation is enhanced by replication blocks or DNA damage; however there is no evidence that Mik1 is a direct substrate of Cds1 or Chk1. Mik1 is a phosphoprotein in vivo, but this modification is not dependent on checkpoint activation [61]. The contribution of Mik1 to the checkpoint arrest of *cdc25(9A)-GFPint* cells was monitored for accumulation of cut phenotypes following HU exposure. *cdc25-GFPint mik1::ura4^+^* cells are much more sensitive than *mik1^+^* cells, but not as sensitive as *rad1-1*. Loss of *cds1^+^* phosphorylation sites on Cdc25 increases the HU sensitivity of cells lacking *mik1* ^+^ (Figure 5A). Western blot analysis shows that the checkpoint defect in *cdc25(9a)-GFPint mik1::ura4^+^* is not due to stabilization of Cdc25(9a)-GFP protein (Figure 5B). Cells of *cdc25(9A)-GFPint* containing a disrupted allele of *wee1* show little accumulation of abnormal mitotic products above the basal level observed prior to HU addition (Figure 5C). Loss of Pyp3 has no effect on the HU sensitivity of *cdc25-GFPint* or *cdc25(9A)-GFPint* (data not shown).

**Figure 6:**
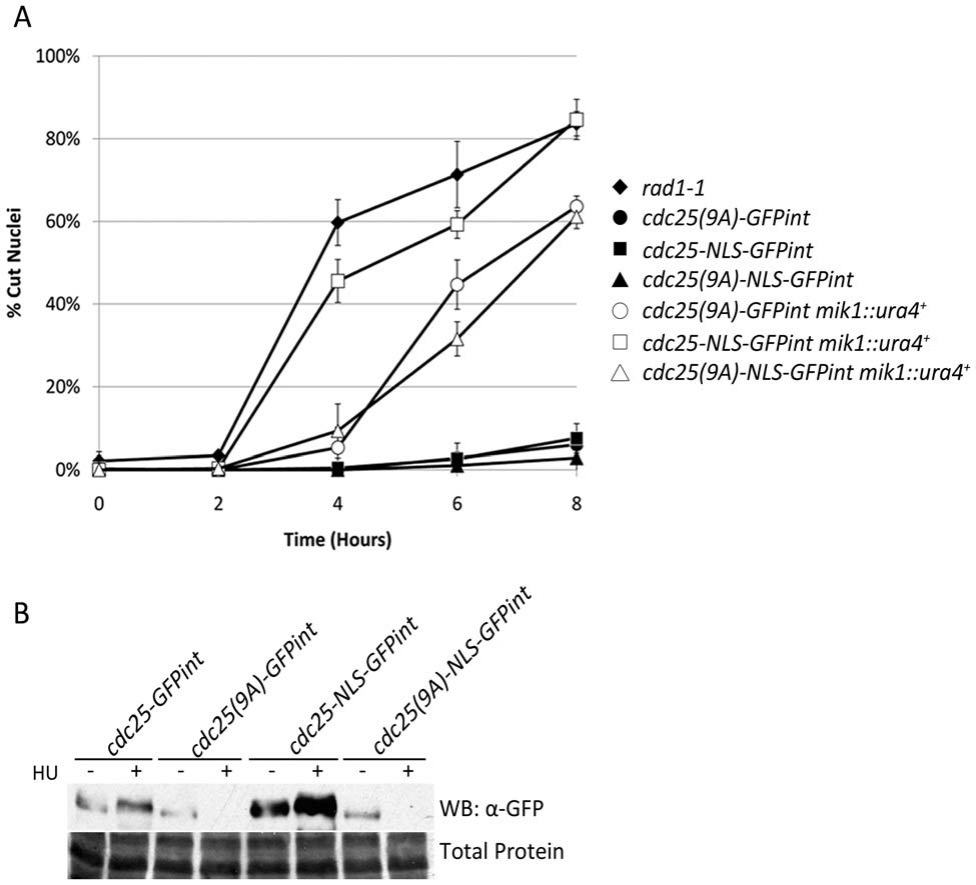
Mik1 is required for replication checkpoint arrest when Cdc25 cannot be exported. **A.** Logarithmic growth phase cultures were sampled prior to and at two hour intervals following exposure to 15 mM HU. Samples were methanol fixed, DAPI stained, and fields of cells photographed and scored for cut nuclei. Error bars represent ±1 s.d. from the mean percent cut phenotype of at least four images containing 50–200 cells from one independent experiment. **B.** Cultures indicated were grown to mid-log phase and exposed to 15 mM HU for 4 hours before collection and processing. Proteins were separated by SDS-PAGE and analyzed by western blot using mouse anti-GFP primary antibody, anti-mouse HRP secondary.

The above experiment indicates that Mik1 is able to prevent mitotic entry under conditions where Cdc25 cannot be inhibited by phosphorylation and 14-3-3 binding. We therefore asked whether Mik1 had a role in the checkpoint proficiency of a strain where Cdc25-GFP is made constitutively nuclear by addition of an SV-40 nuclear localization signal (NLS). A similar strain was utilized to demonstrate that cytoplasmic localization of Cdc25 is not required for checkpoint proficiency [49]. *cdc25-NLS-GFPint* is not significantly more sensitive to HU than *cdc25-GFPint* when *mik1^+^* is present (Figure 6). However in the absence of *mik1*, *cdc25-NLS-GFPint* shows a profound checkpoint defect, approaching the severity of *rad1-1*. The sensitivity of *cdc25-NLS-GFP* can be reduced by mutagenesis of the nine S/T Cds1 phosphorylation sites (creating *cdc25(9A)-NLS-GFP*) showing that the instability induced by 9A mutations is dominant to the advancement of mitosis caused by forcing Cdc25 to be nuclear.

## Discussion

### Cdc25(9A)-GFP native promoter integrant does not have a cell cycle phenotype

It has previously been reported that Cdc25 is hyperphosphorylated and interacts with Rad24 during interphase [62]. This was thought to be consistent with the finding that cells lacking Rad24 divide at a length somewhat smaller than wildtype [51]. A similar phenotype can be seen when Cdc25 is forced into the nucleus by addition of an SV-40 nuclear localization signal [16], [49]. Both loss of *rad24* and addition of an exogenous NLS to Cdc25 cause enhanced Cdc25 nuclear localization, as is observed in Cdc25(9A)-GFP. Thus, it is surprising that cells expressing Cdc25(9A)-GFP divide at a size identical to wildtype. However, the enhanced nuclear localization of Cdc25 may be due to mislocalization of another Rad24 binding partner, rather than from disrupting the Cdc25-Rad24 interaction per se.

Previous reports indicate that features in the 5′ UTR of Cdc25 act to limit translation when nutrients are limited thus tying Cdc25 accumulation to the overall rate of translation in the cell [57]. Even so, it is surprising that this translates into a many fold difference in Cdc25 protein expression level in YEA vs minimal media while the decrease in generation time is about 20% (2.5 hours in YEA, 3 hours in EMM). This is particularly interesting since Cdc25 is traditionally thought of as a dose-dependent mitotic inducer. Overexpression of Cdc25 from a strong *nmt1* promoter causes a drastic decrease in cell size. However, under physiological regulation a cell divides at a very similar size in conditions where Cdc25 concentration is vastly different, such as where cells are grown in rich or minimal media. This observation supports the model of the cell size checkpoint where, rather than being quantitatively rate limiting for mitotic initiation, Cdc25 is discretely activated only when the cell has achieved a threshold cell size [63].

### Cdc25(9A)-GFP overexpression causes a DNA replication checkpoint defect

Re-examination of the results of Zeng and Piwnica-Worms [50] suggest that their conclusions were influenced by over-expression of the mutant Cdc25(9A) protein. Protein levels in our Cdc25-GFP native promoter integrant are approximately 10 fold lower than the expression from the *nmt81* promoter. At the permissive temperature of 30°C *cdc25-22^ts^* cells expressing an empty vector do not accumulate *cut* nuclei following HU exposure [50]. Thus the mitotic catastrophe seen in cells expressing *pREP81-cdc25(9A)* can be considered a dominant negative phenotype attributable to high protein expression. The results shown here highlight the importance of using chromosomal integrations and native protein expression levels. This is particularly important with proteins such as Cdc25, well known to have a dose dependent effect. It is likely that expressing *cdc25(9A)* from the *pREP81* promoter overwhelmed the ability of the cell to destroy the phosphatase, resulting in bypass of the replication checkpoint in the presence of HU.

### Mik1 is required to enforce the replication checkpoint when Cdc25 cannot be exported or phosphorylated

Mik1 is involved in repressing mitotic entry from S-phase during unperturbed growth [61] or following HU exposure [64] by maintaining Cdc2 Y15 phosphorylation. Here we show that in cells unable to inhibit Cdc25 by phosphorylation at the 9 S/T sites in its regulatory domain are still able to maintain a checkpoint arrest through Mik1, with Wee1 playing a minor role. Although Mik1 is involved in the response to replication arrest, it does not appear to be directly phosphorylated by the Cds1 or Rad3 kinases [22]. However, accumulation of Mik1 following HU exposure requires a functional checkpoint response [65]. Mik1 is regulated by the *MluI* cell-cycle-box binding factor (MBF) complex, resulting in G1/S specific expression [41]. MBF members Cdc10 and Rep2 are both activated, and the MBF repressor protein Nrm1 inhibited, by Cds1 mediated phosphorylation [66]–[69].

Mik1 is required to prevent mitotic initiation in cells expressing Cdc25-NLS, where Cdc25 is forced to remain nuclear following replication checkpoint activation. The checkpoint proficiency of Cdc25-NLS-GFP was a key observation in the model presented by [49]. That is, the cytoplasmic relocalization and segregation from Cdc2, is not required for replication and DNA damage checkpoint function. This model predicts that phosphorylation and 14-3-3 binding to Cdc25-NLS is sufficient to inhibit its phosphatase activity and prevent Cdc2 Y15 dephosphorylation even if Cdc25 remains localized to the nucleus.

Cds1 interacts with and phosphorylates Wee1 [17]. However, Wee1 appears to play a minor role in arrest following HU exposure in cells expressing Cdc25(9A)-GFP. Thus, the results presented here are consistent with previous studies which showed that cells lacking *cdc25* in a *wee1-50^ts^* background are resistant to HU [70].

### Cdc25 phosphorylation mutants are unstable following replication arrest

The rapid degradation of Cdc25(9A)-GFP following HU treatment suggests that one of the functions of Cdc25 phosphorylation is to protect the phosphatase from proteasomic degradation during DNA replication checkpoint arrest. These results indicate that phosphorylation/14-3-3 binding is required for the stockpiling response observed by Kovelman and Russell [56]. Cdc25(9A)-GFP shows a higher rate of turnover in promoter shutoff experiments suggesting that either phosphorylation or 14-3-3 binding has a stabilizing function during unperturbed growth as well.

Whether Cdc25 is stabilized by constitutive low level phosphorylation and 14-3-3 binding during interphase is not certain. Rad24 and Cdc25 have been reported to interact in unperturbed cells [62]. However, the lack of a cell cycle defect in *cdc25(9A)-GFPint* argues that such an interaction is not required during normal growth.

### Cdc25 phosphorylation is required to maintain viability following HU treatment

Although cdc25(9A)-GFPint fails to accumulate an increased number of cut nuclei within 8 hours of HU exposure, the growth of this strain on media containing HU is clearly inhibited relative to wildtype. The role of these phosphorylation sites in maintaining viability following this arrest is not clear; absence of phosphorylation however does lead to degradation following arrest. Kovelman and Russell [56] hypothesized that stockpiling may be adaptive to allow rapid re-entry into the cell cycle once the checkpoint arrest is lifted. Alternately, residual amounts of Cdc25(9A)-GFP may cause a subpopulation of cells to eventually leak through the checkpoint leading to a slow, cumulative loss of viability.

## Materials and Methods

### General cell culture techniques

*S. pombe* cultures were grown in Edinburgh Minimal Media (EMM) or Yeast Extract supplemented with adenine (YEA) [71]. In experiments where expression was regulated by the *nmt1* promoter, 15 µM thiamine was used for repression. Cell density was measured using a Multisizer 3 Coulter Counter (Beckman Coulter). Genetic crosses were conducted on Sporulation Agar (SPA) according to Gutz et al. [72] Plasmids were introduced into *S. pombe* cells by electroporation using a BioRad Gene Pulser as described by Prentice [73]. A list of strains used in this study is presented as Table 1.

**Table 1:** Strains used in this study.

Strain Number	Genotype	Source
Q3676	*ura4-D18 leu1-32 h^−^*	Lab Collection
Q3677	*ura4-D18 leu1-32 h^+^*	Lab Collection
Q1975	*cdc25::ura4^+^ cdc2-3w ura4-D18 leu1-32*	Russell and Nurse, 1986
Q2016	*cdc25-GFPint cdc25::ura4^+^ ura4-D18 leu1-32 h^−^*	Chua et al, 2002
Q3792	*cdc25(9A)-GFPint cdc25::ura4^+^ ura4-D18 leu1-32 h^+^*	This Study
Q250	*972 h^−^*	Lab Collection
Q3799	*cdc25-GFPint rad1-1 ura4-D18 leu1-32*	This Study
Q3832	*pREP81-cdc25-GFP cdc25-22 leu1-32*	This Study
Q3898	*pREP81-cdc25(9A)-GFP cdc25-22 leu1-32*	This Study
Q3780	*pREP81-cdc25(9A) cdc25-22 leu1-32*	This Study
Q3977	*cdc25-GFPint cdc25::ura4^+^ wee1::ura4^+^ ura4-D18 leu1-32*	This Study
Q3978	*cdc25(9A)-GFPint cdc25::ura4^+^ wee1::ura4^+^ ura4-D18 leu1-32*	This Study
Q3870	*cdc25-GFPint cdc25::ura4^+^ mik1::ura4^+^ ura4-D18 leu1-32*	This Study
Q3866	*cdc25(9A)-GFPint cdc25::ura4^+^ mik1::ura4^+^ ura4-D18 leu1-32*	This Study
Q2021	*cdc25-NLS-GFPint cdc25::ura4^+^ ura4-D18 leu1-32*	Chua et al, 2002
Q3810	*cdc25(9A)-NLS-GFPint cdc25::ura4^+^ ura4-D18 leu1-32*	This Study
Q3942	*cdc25-NLS-GFPint cdc25::ura4^+^ mik1::ura4^+^ ura4-D18 leu1-32*	This Study
Q3943	*cdc25(9A)-NLS-GFPint cdc25::ura4^+^ mik1::ura4^+^ ura4-D18 leu1-32*	This Study
Q3974	*cdc25-GFP rad24::ura4^+^ ura4-D18 leu1-32*	This Study
Q3882	*cdc25-GFPint cdc25::kanMX6 ura4-D18 leu1-32*	This Study
Q3883	*cdc25(9A)-GFPint cdc25::kanMX6 ura4-D18 leu1-32*	This Study
Q3884	*cdc25(12A)-GFPint cdc25::kanMX6 ura4-D18 leu1-32*	This Study

### Cloning and genomic integration

A PCR fragment containing the 1869 bp *cdc25* ^+^ open reading frame (ORF) and 1551 bp of 5′ upstream sequence was amplified from wildtype genomic DNA using primers *cdc25cf1* (5′-ACGCCTGCAGTCCGAGTTTAACAAGACAACTGGC-3′) and *cdc25gc3* (5′-ACGCGTCGACGAAAATCTTCTAAGTGTAGAGAGGGAATGCA-3′), and digested with *PstI* (Promega) and *SalI* restriction enzymes (Promega). The *nmt1* promoter of *pREP1-GFP* was excised with *PstI* and *SalI* and cut vector and insert were ligated to create *pcdc25-GFP*. A vector containing a *cdc25(9A)* allele, where 9 of 12 putative Cds1 serine/threonine phosphorylation sites were substituted with alanine was acquired from Helen Piwnica-Worms in the form of *pREP81-cdc25(9A)* where site-directed mutagenesis was used to make the following substitutions: S99A, S148A, S178A, S192A, S204A, S206A, T226A, S234A and S359A [50]. An 845 bp SplI/BglI fragment of the *cdc25(9A)* open reading frame containing all 9 of the alanine substitutions was excised and ligated into the *pcdc25-GFP* vector, likewise cut with SplI/BglI, creating the vector *pcdc25(9A)-GFP*. To create *pREP81-cdc25(9A)-GFP*, the *cdc25(9A)* ORF from *pREP81-cdc25(9A)* vector was excised using *NdeI* and *SalI* and ligated into *pREP81-GFP*.

Plasmids containing *cdc25-GFP* or the various phosphorylation site mutants were transformed into *cdc25::ura4^+^ cdc2-3w ura4-D18 leu-32 (Q1975)* and stable integrants selected. Integration at the *cdc25* locus was confirmed genetically by crossing to *ura4-D18 leu1-32* (Q3676 or Q3677) and observing 2∶2 segregation between leucine prototrophs and auxotrophs, co-segregation of leucine and uracil prototrophs, and the lack of *cdc* ^−^ spores. The presence of all 9 S/T to A substitutions in the *cdc25(9A)-GFPint* strain was confirmed by recovery of the chromosomal integrant by PCR and sequencing.

### Microscopy

Bright field and DAPI/methyl blue images were captured with a Leica DMRB fluorescence microscope (Leica Micro-systems) equipped with a high performance CCD camera (Cooke SensiCam) and analysed using Slidebook (Intelligent Imaging Innovations). DAPI staining was carried out in100 mM Tris-HCl pH 7.5 following cold methanol fixation [74]. GFP florescence images were captured using a Zeiss Imager.Z1 equipped with a Hamamatsu ORCA-ER CCD camera.

### Preparation of total protein from *S. pombe*

Cells were grown in liquid culture to mid-logarithmic phase. A 25 ml volume was chilled to 0°C in a 50 ml conical bottom tube by addition of 20 ml of crushed, frozen media. The addition of ice gave rapid cooling and was essential in order to prevent phosphorylation of Cdc25 due to cold shock and centrifugation in response to activation of the stress activated map kinase pathway [75], [76]. Cells were then collected at 0°C, resuspended in 1 ml of Stop Buffer (150 mM NaCl, 50 mM NaF, 10 mM EDTA, 1 mM NaN_3_) [56], briefly centrifuged at 13000 rpm and the supernatant removed. The resulting pellet was frozen in dry ice and stored at −80°C until processed.

Cell pellets were thawed in 150 µl of modified SUME buffer (1% SDS, 8 M urea, 10 mM MOPS pH 6.8, 10 mM EDTA) [77] containing 1× Complete Protease Inhibitor Cocktail (Roche) and 1 mM PMSF. Cells were broken by agitation in the presence of acid-washed glass beads (0.5 mm diameter, BioSpec) using a bead beater (BioSpec Products, Barttesville, OK, USA) until 80–100% breakage was achieved. The bead slurry was centrifuged briefly, mixed gently with 150 µl of fresh SUME buffer and the lysate transferred to a fresh microfuge tube. After centrifugation at 13,000 RPM for 10 minutes at 4°C to remove debris the cleared supernatant was transferred to a fresh tube. Protein concentration was determined using a BioRad Protein Assay Kit. 4× Laemmli loading dye (200 mM Tris-HCl pH 6.8, 8% SDS, 40% glycerol, 0.33% β-mercaptoethanol, bromophenol blue) was added to the lysates and the samples were heated to 100°C for five minutes.

### SDS-PAGE and western blotting

Proteins were separated using SDS-PAGE on 6% acrylamide gels and electrophoretically transferred to PVDF membrane (Perkin Elmer, Wellesley, MA, USA) for western blotting. Non-specific antibody binding was inhibited by incubating the membrane in blocking buffer (5% non-fat skim milk powder, 0.05% Tween-20 in 1× TBS) for thirty minutes. This was followed by three five minute washes in TBS containing 0.5% Tween-20 then incubation with a 1∶2000 dilution of mouse anti-GFP monoclonal (Roche Molecular Biochemicals) in blocking buffer for one hour followed by three washes of TBS 0.05% Tween-20. Membranes were incubated with horseradish peroxidase-conjugated goat anti-mouse secondary antibody (Santa Cruz Biotechnology, CA, USA) diluted 1∶2000 in blocking buffer for 20 minutes followed by six washes in TBS with 0.2% Triton-X100 and two washes with TBS lacking detergent. Membranes were then treated with chemiluminescence reagents (GE Health Sciences) and exposed to x-ray film (Kodak X-Omat Blue). Following western blotting, membranes were stained with Coomassie Brilliant Blue for demonstration of equal protein loading. Due to the use of 6% poly-acrylamide gels and the running duration required to resolve the electrophoretic mobility differences resulting from Cdc25 phosphorylation we were unable to use common loading controls such as Cdc2, Actin or Tubulin as these proteins were run off the gel. A recent paper has shown a linear relationship between protein loading and Coomassie staining intensity [78].
